# Crack Development and Healing in Guar Gum Polymer–Modified Silty Clay Under Natural Wetting–Drying Cycles

**DOI:** 10.3390/polym18010013

**Published:** 2025-12-20

**Authors:** Wanxin Hou, Xiyan Jiang, Xu Wang, Dameng Wang, Daye Du

**Affiliations:** 1 College of Civil Engineering, Hebei University of Architecture, Zhangjiakou 075000, China; hwx2023201107@hebiace.edu.cn (W.H.); wx2024201113@hebiace.edu.cn (X.W.); wdm2024201109@hebiace.edu.cn (D.W.);; 2 Hebei Provincial Engineering Technology Innovation Center for Transportation Infrastructure in Cold Regions, Zhangjiakou 075000, China

**Keywords:** wetting–drying cycles, guar gum polymer, connectivity coefficient, crack depth ratio, microstructure

## Abstract

This study investigates the evolution characteristics of fissure networks in cohesive soils under wetting–drying cycle conditions with varying guar gum content. Four wetting–drying cycles were conducted under outdoor natural conditions, with real-time monitoring of changes in the surface crack network during drying and wetting. Geometric parameters—including surface crack density, width, connectivity coefficient, shape coefficient, and crack depth ratio—were quantitatively analyzed using digital image processing software. Scanning electron microscopy (SEM) and X-ray diffraction (XRD) were employed to reveal the mechanisms of microstructural improvement. Results indicate that as wetting–drying cycles increase, the fracture network progressively simplifies, with fracture density and fractal dimension decreasing while fracture width increases. The incorporation of guar gum reduced the crack depth ratio to approximately 0.62 times that of undamaged soil. The average crack width decreased from 2.69 mm to 2.16 mm during the fourth wet-dry cycle, whilst the connectivity coefficient and shape coefficient stabilized. SEM analysis indicated that guar gum promoted “bonded bridging” structures between soil particles, while XRD results confirmed no alteration in the mineral composition of the soil. The study demonstrates that the addition of guar gum enhances soil crack resistance and stability, providing theoretical support for the ecological protection of clayey slopes.

## 1. Introduction

In arid environments, soil dehydration and contraction often lead to the formation of surface cracks, significantly reducing soil strength, water retention, and stability. Cracked soil exhibits greater compressibility than intact soil, leading to various engineering geological issues. Moreover, the crack network’s strength is notably weakened [1,2]. The geometric morphology of this crack network plays a critical role in controlling solute migration within the soil, which significantly increases its permeability [3,4]. In real-world engineering applications, crack formation adversely affects foundation treatment for structures, subgrade treatment for highways and railways, and slope stability and reinforcement, thereby reducing or even compromising their waterproofing functionality [5,6,7].

Both domestic and international research have explored factors influencing soil crack network morphology, including soil thickness [8], interface roughness [9], temperature and humidity [10], and the wetting-drying cycle. Among these, wetting-drying cycles are among the most common natural factors affecting soil, making them an essential subject of study. Existing studies have shown that wetting-drying cycles lead to deformation in soils due to drying and swelling, altering their internal structure and significantly affecting the geometry of the crack network [11]. For instance, Bu et al. [12] examined the synergistic effects of wetting-drying cycles on sisal fibre and polyacrylamide, finding improvements in soil water retention and crack resistance. Tang et al. [13] investigated crack development in clayey soils under different wetting-drying conditions, noting that more cycles led to more pronounced cracks and greater secondary crack expansion. Similarly, C. S. Tang et al. [14] found that the number of crack segments increased with the number of cycles, thereby reducing soil brittleness. Guo et al. [15] studied the effects of wetting-drying cycles on jute fibre-modified clay, revealing that jute fibres effectively inhibit crack propagation, with increased fibre content reducing crack rate and width. M. Julina and T. Thyagaraj [16] explored the combined effects of wetting-drying cycles and salt solutions on drying cracks in compacted clay, visualizing and quantifying these cracks using XCT imaging.

As crack research evolves from qualitative descriptions to quantitative analysis, image acquisition and digital image processing technologies have increasingly become crucial technical approaches for quantifying crack development. Li Ke et al. [17], Hu Dongxu et al. [18], and Su Lijun [19] extracted surface cracks in soil using PCAS, CIAS, and MATLAB2022b, providing data support for crack evolution studies. However, existing research has primarily focused on the cracking stage, lacking quantitative descriptions of the wetting-healing process and structural stabilization mechanisms. Moreover, most studies rely on controlled indoor conditions, making it challenging to reflect the coupled effects of temperature, humidity, wind speed, and other factors in natural environments. Particularly in the field of biopolymer-modified soils, research on the temporal evolution of crack opening, closing, healing, and connectivity changes under natural wetting-drying cycles remains insufficient. Among numerous biopolymers, guar gum—a low-cost, widely available, and environmentally friendly biopolymer—is extensively used in soil improvement due to its excellent modification effects.

Building on prior research on single dehumidification stages [20], this study conducted four dry-wet cycle tests under natural environmental conditions to elucidate the developmental, healing, and regenerative evolution patterns of fissures in guar gum-modified silty clay under cyclic wetting and drying. To thoroughly analyze changes in the fissure network structure, novel metrics including the fissure connectivity coefficient, fissure depth ratio, and shape coefficient were introduced, establishing a quantitative framework for fissure network evolution. Concurrently, scanning electron microscopy (SEM) and X-ray diffraction (XRD) analyses were employed to investigate the microstructural mechanisms of guar gum-modified soil. This research aims to elucidate the crack-resistant and self-healing properties of biopolymer-modified soil under wet-dry cycling conditions, thereby providing theoretical foundations for the ecological protection of cohesive soil slopes.

## 2. Experimental Materials and Methods

### 2.1. Experimental Materials

(1) Soil samples

**Figure 1:**
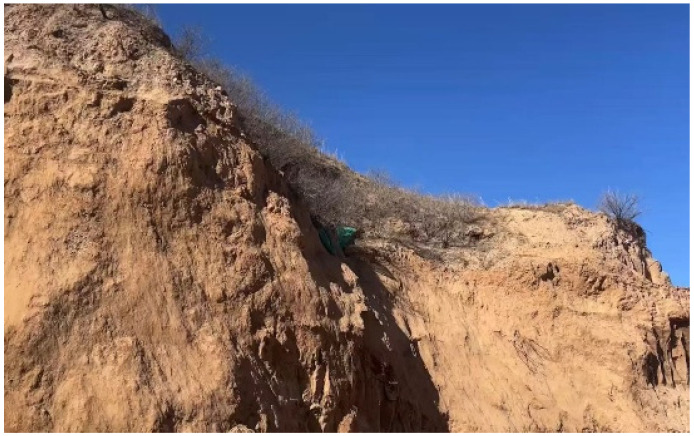
A bare soil slope in Zhangjiakou City.

The soil samples used in the experiment were collected from a slope engineering site in Zhangjiakou City, Hebei Province, China, as shown in Figure 1. After collection, the samples were dried, crushed, and sieved through a 2 mm sieve according to the ‘Standard for Soil Testing Methods’ [21] (GB/T50123-2019). The basic physical properties of the soil are listed in Table 1. Based on the ‘Code for Design of Building Foundations’ (GB 50007-2011) [22], the soil was classified as silty clay.

(2) Biopolymers

**Figure 2:**
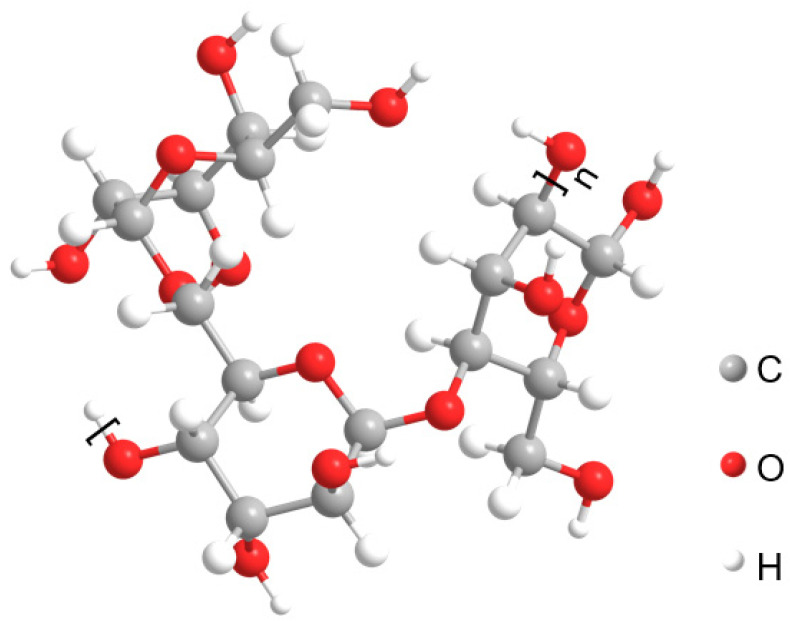
Morphological and molecular structure of guar gum.

The experiment used industrial-grade guar gum, a pale yellow, powdered biopolymer (Figure 2) extracted from guar beans. It is an odourless, non-toxic polysaccharide with a high molecular weight, primarily produced in India, Brazil, and South Africa, with India being the leading global producer. Guar gum is one of the most economically viable sources of galactomannan. The molecular formula is shown in Figure 2. It is a water-soluble natural polysaccharide with 95% purity and a molecular weight of up to 5000 kDa. The molecular structure consists of a leading chain of β-D-mannopyranose units, with α-D-galactopyranose units attached to the main chain, with a galactose-to-mannose ratio of approximately 1:2 [23,24]. The four hydroxyl groups on the side-chain galactose units can participate in esterification or etherification reactions. Due to steric hindrance, the primary hydroxyl group in the hydroxymethyl group is the most reactive, while the hydroxyl groups on the main-chain mannose units also exhibit reactivity. The numerous hydroxyl groups (-OH) on its molecular chain give guar gum strong hydrophilicity, allowing it to form high-viscosity colloidal solutions in water and physically adsorb onto the hydrogen-bonded surfaces of soil particles.

(3) Crack Depth Measurement Instrument

**Figure 3:**
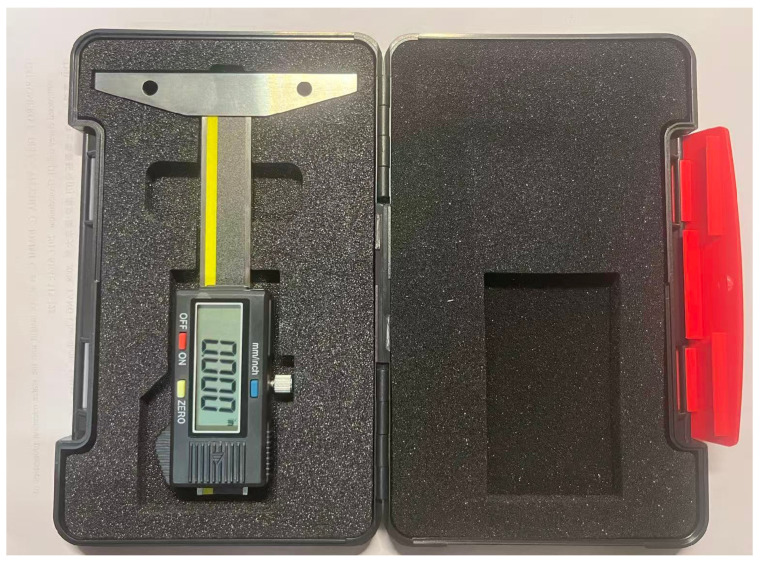
High-Precision Digital Depth Gauge with Fine-Spline Rod.

The depth of cracks was measured using a high-precision digital depth gauge with a fine rod manufactured in Shanghai, China (as shown in Figure 3). During measurement, multiple measurement points were selected within the crack area. At each measurement point, the gauge rod was aligned with the crack, ensuring its axis was perpendicular to the soil surface. The rod was slowly lowered to the bottom of the crack, avoiding contact with the crack walls. Repeat the measurement three times at each point, recording the maximum value as the depth for that point to enhance data reliability. Document the depth values for all measurement points. The maximum value among all measurement points is ultimately selected as the maximum crack depth for the specimen.

### 2.2. Sample Preparation

Findings from prior research reveal that the guar gum content (powder/dry soil mass ratio) was set at 0%, 0.5%, 1%, 1.5%, and 2% [20]. Modified soil samples were prepared using a powder-premixing method: the calculated amount of guar gum was uniformly mixed with dry clay sieved through a 2 mm mesh. Water was then added in a specific mass, thoroughly stirred, and sealed for 24 h. Cylindrical stainless steel containers (Ø250 × 90 mm) were used to prepare samples for the wet-dry cycle. The soil was compacted into these containers using a compaction method, achieving a degree of compaction of 85%, an optimal moisture content of 18.1%, and a thickness of 6 cm.

### 2.3. Test Procedure

To investigate the evolution of soil cracks during the wetting-drying cycle, four tests were conducted in sequence, wetting followed by drying. During wetting, water was uniformly sprinkled onto the sample surface until the moisture content reached 26.83% (near saturation). After adding water, the sample was left to stand for 24 h to ensure uniform moisture distribution.

For drying, the saturated samples were placed outdoors. Moisture levels were measured daily using weight-based methods, with at least eight measurements per day. The drying process was considered complete when the moisture content changed by less than 0.1% over 72 h.

Photography was conducted using a Sony FDR-AX45A camera (resolution 1440 × 1080) from Shanghai, China, to monitor crack morphology and humidity variations. In the early stages, crack changes were more pronounced (especially under high temperatures and intense evaporation in midsummer, or low temperatures and strong winds in early winter). Therefore, photos were taken eight times daily at 2 h intervals to capture the dynamic crack development. After day six, crack growth became more stable. Based on prior observations, the photography frequency was adjusted to 5 times per day at 4 h intervals to track crack stabilization. Each drying process lasted 16 days, totaling approximately 4 months across all four cycles. Photography was completed once the moisture content stabilized at the end of each drying phase.

The above wetting, drying, and photography process was repeated four times to match the climatic conditions of midsummer, late summer, autumn, and early winter, ensuring data comparability across the cycles and accurately quantifying the evolution of cracks under the combined effects of different seasons and wetting-drying cycles.

### 2.4. Meteorological Data

**Figure 4:**
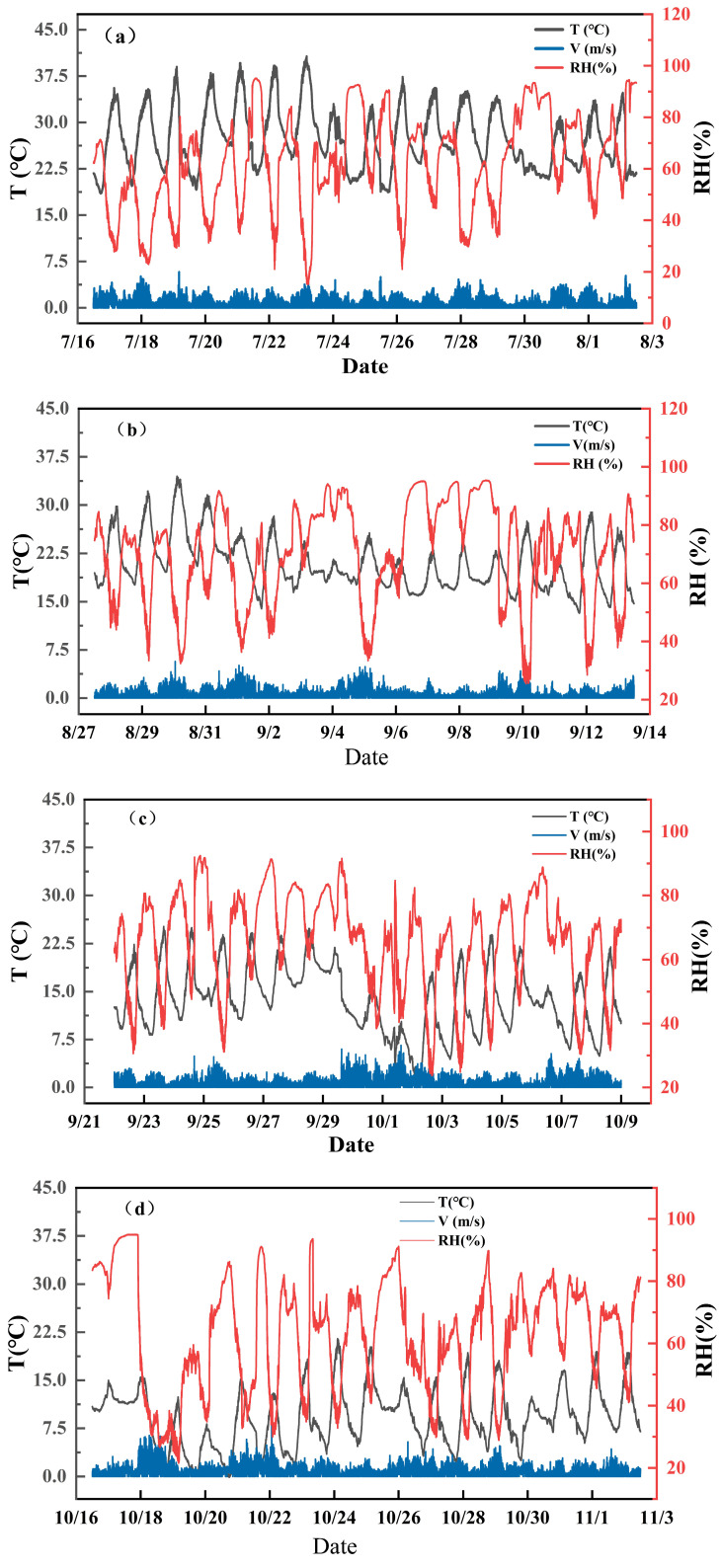
A graph showing meteorological changes during four wetting–drying cycles. (**a**) Weather variation curve for the second dry-wet cycle, (**b**) Weather variation curve for the second dry-wet cycle, (**c**) Weather variation curve for the third dry-wet cycle, (**d**) Weather variation curve for the fourth dry-wet cycle.

Building upon preliminary experiments, this study extended the monitoring period to enhance the analysis of the coupled effects of climatic fluctuations on crack healing and regeneration processes. utilizing the PC-9XQH automatic weather monitoring system, environmental parameters, including temperature, humidity, and wind speed, were acquired in real time. The experiment comprised four wetting–drying cycle phases corresponding to typical climatic periods: midsummer, late summer, autumn, and early winter. Meteorological data were automatically collected at one-minute intervals. The start and end dates of each phase, along with their primary meteorological characteristics, are detailed in Table 2. The trends in temperature, humidity, and wind speed across the four cycles are illustrated in Figure 4. These data reveal pronounced temporal variations in meteorological conditions: significant temperature fluctuations, marked variations in humidity, and substantial differences in wind speed across periods, reflecting typical seasonal climatic patterns. It should be noted that meteorological data is employed solely for the qualitative presentation of temporal variations in the wet-dry cycle within the natural climatic context, to aid understanding of the phased development and healing processes of fissures. It is not utilized for quantitative causal analysis of the relationship between temperature and humidity fluctuations and fissure behaviour. Changes in relevant variables are provided merely as contextual information.

### 2.5. Digital Image Processing

Image recognition and parameter extraction methods draw upon prior research, employing Python2023-based custom programmes and PCAS for automated identification and feature quantification of crack images. To accommodate the temporal variations across the four wetting–drying cycles studied, the algorithm incorporates key crack metrics—connectivity coefficient, shape coefficient, and crack depth ratio—beyond the baseline workflow. This enables dynamic comparative analysis of crack networks between wet and dry cycle phases. For detailed image processing procedures and fundamental parameter definitions, refer to Refs. [25,26,27,28,29].

## 3. Experimental Results and Discussion

### 3.1. Development Process of the Crack Network in the First Wetting-Drying Cycles

#### 3.1.1. First Drying Process

**Figure 5:**
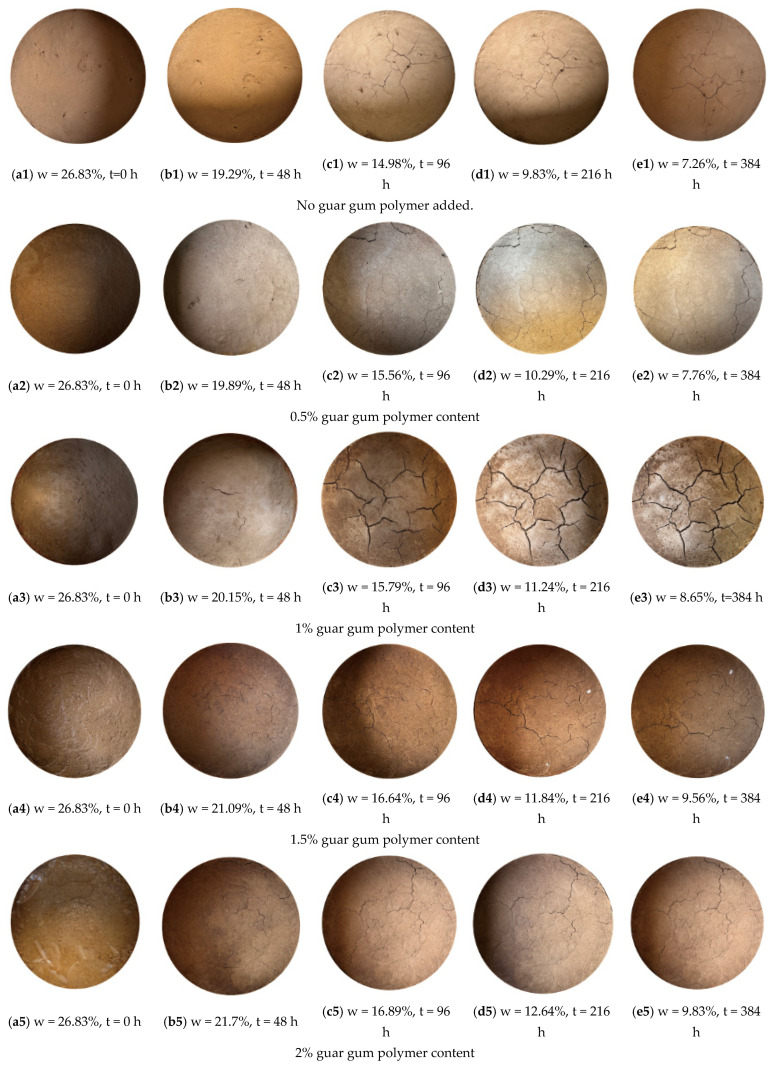
Development process of the crack network during the first wetting-drying cycles. (**a1–e1**) represent the crack evolution images without guar gum polymer. (**a2–e2**) represent the crack evolution images with 0.5% guar gum polymer. (**a3–e3**) represent the crack evolution images with 1% guar gum polymer. (**a4–e4**) represent the crack evolution images with 1.5% guar gum polymer. (**a5–e5**) represent the crack evolution images with 2% guar gum polymer.

Figure 2 illustrates the development and evolution of cracks in five sample groups during the initial drying process. Regular photography of the samples recorded the dynamic crack formation, showing that cracks developed sequentially. Compared to plain soil, during the initial drying phase (i.e., after 24 h of drying, as shown in Figure 5(b1)), the sample surface exhibited only fine, discrete cracks, without typical T-shaped or Y-shaped structures, and no through cracks were observed. After 24 h, as the moisture content decreased, the cracks gradually expanded. In contrast, the guar gum polymer-modified samples formed penetrating T-shaped elongated cracks during the initial drying stage (Figure 5(b2–b5)) due to the guar gum polymer’s effect on the soil’s shrinkage properties, leading to more pronounced cracking. As the moisture content continued to decrease, the cracks became more stable and evolved into minor, Y-shaped cracks. In the late drying stage (after 240 h, as shown in Figure 5(e2–e5)), the samples contracted further. The reduction in guar gum polymer adhesion caused the soil to break into several blocks, stabilizing the crack morphology.

#### 3.1.2. First Wetting Process

**Figure 6:**
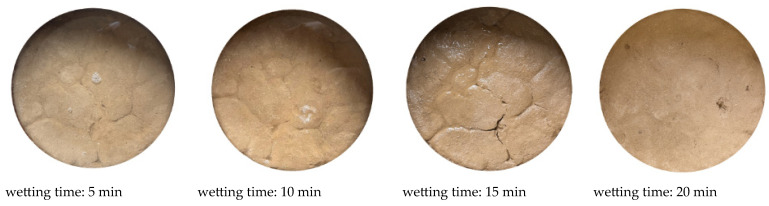
Diagram of the crack healing process during the first wetting.

After the first drying process, the soil sample surface is uniformly watered in layers until it is fully saturated. Using 1% guar gum polymer as an example (Figure 6), cracks on the soil surface tend to heal as the moisture content increases. Crack closure generally occurs through two mechanisms: first, hydration expansion of clay particles, and second, mechanical closure. Hydrophilic clay minerals absorb water, forming a hydration film on the particle surfaces, which leads to soil volume expansion and promotes crack closure. This process is typically accompanied by soil uplift at the original crack location [30,31].

The second mechanism is the collapse-filling of crack edges. As the soil absorbs moisture, the cohesive force within the soil matrix decreases, leaving the crack edges unsupported and allowing them to open further. The loss of support leads to soil collapse, which gradually fills the crack space, closing it. This type of closure is often accompanied by the formation of a ‘healing ridge’ at the crack site [32]. The ‘healing ridge’ morphology is widely observed in the crack-healing regions of this experiment, indicating that the collapse-filling mechanism dominates the crack-healing process during hydration. This phenomenon is closely related to the characteristics of the experimental soil. Since the soil used in this study is a clayey silt with minimal expansivity, the hydration expansion mechanism has a significantly reduced effect, thereby highlighting the decisive role of the collapse-filling mechanism in crack healing.

### 3.2. Characteristics of Crack Morphology Evolution Under the Influence of the Second to Fourth Wetting-Drying Cycles

**Figure 7:**
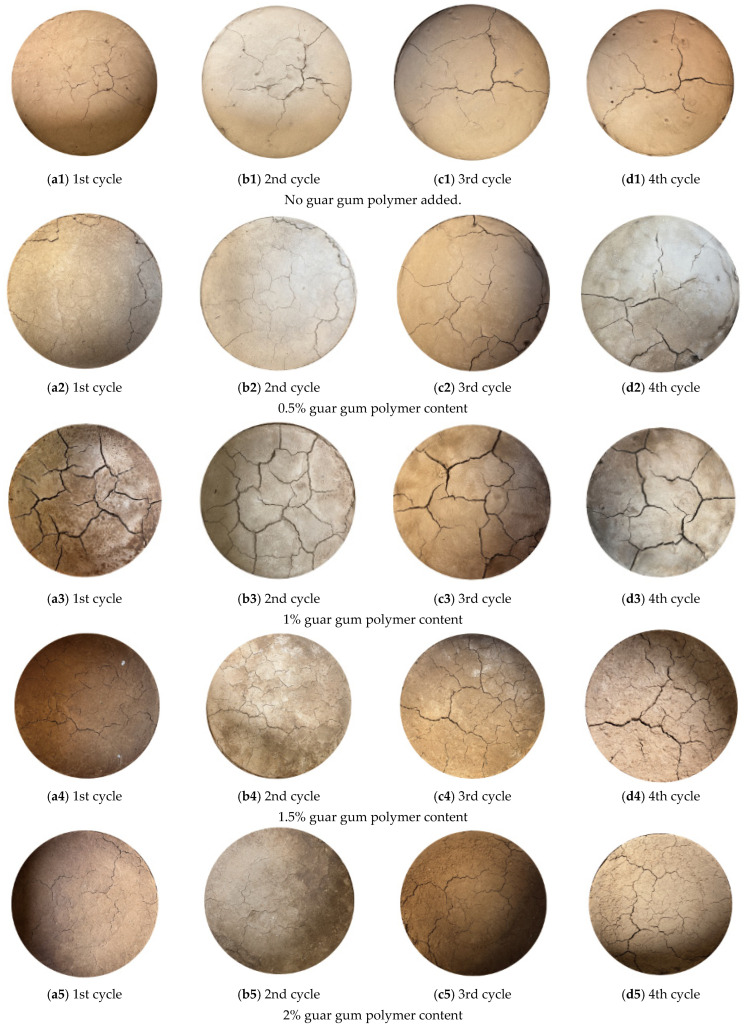
Evolutionary characteristics of crack morphology under the influence of the second to fourth wetting-drying cycles. (**a1–d1**) represent the final images of the first, second, third, and fourth cycles, respectively, without guar gum polymer. (**a2–d2**) represent the final images of the first, second, third, and fourth cycles, respectively, with 0.5% guar gum polymer. (**a3–d3**) represent the final images of the first, second, third, and fourth cycles, respectively, with 1% guar gum polymer. (**a4–d4**) represent the final images of the first, second, third, and fourth cycles, respectively, with 1.5% guar gum polymer. (**a5–d5**) represent the final images of the first, second, third, and fourth cycles, respectively, with 2% guar gum polymer.

After completing the first wetting–drying cycle, the subsequent three cycles were executed sequentially. The evolution of cracks after the first to fourth drying processes is shown in Figure 7. As seen in the figure, after the first cycle (Figure 7(a1–a5)), clods of plain soil exhibit well-defined boundaries with small crack widths. In contrast, the cracks in the guar gum polymer-modified samples are more developed, often forming T-shaped or Y-shaped intersections. After the second cycle (Figure 7(b1–b5)), the soil boundaries become irregular, the crack width increases, the number of crack nodes decreases, and the cracks appear in the same positions as those from the first drying cycle. Following the third cycle (Figure 7(c1–c5)), compared to the first cycle, the crack locations are less distinct, the soil block boundaries become more irregular, and the crack network simplifies. As the number of wetting-drying cycles increases to the fourth cycle (Figure 7(d1–d5)), the crack morphology becomes similar to that of the third cycle, indicating that crack development has gradually stabilized.

Additionally, it was observed that soil samples with a guar gum polymer content of ≤1% exhibited larger fragmentation areas, whereas those with a guar gum polymer content of >1% exhibited smaller fragmentation areas. The fragmentation areas of all five guar gum polymer content levels corresponded with the crack traces formed after the first cycle. By comparing the orientation of cracks with the distribution of ‘healing ridges’, it was found that most new cracks redeveloped along the ‘healing ridges’ formed during the first wetting-drying cycle. This pattern is consistent with crack regeneration in clay soils [33]. The recurrence of cracks at their original locations is due to the structural characteristics of the ‘healing ridges’. These regions, formed from the healing of cracks in the previous cycle, have a looser structure and tend to lose water and contract during subsequent drying, thereby weakening them relative to the surrounding matrix. This results in stress concentration and premature failure. Therefore, the crack network generated from the second to the fourth cycles is essentially the same as the one formed in the first cycle. Furthermore, compared to the initial drying stage, the intricacy of the cracks decreases as the number of wetting–drying cycles increases.

### 3.3. Quantitative Analysis of Crack Morphology Evolution

#### 3.3.1. Effect of Wetting–Drying Cycles on Desiccation Time

Desiccation time refers to the duration required for desiccation during each wetting–drying cycle, a parameter that directly reflects the strength of soil water-holding capacity. As previously noted, guar gum polymer significantly enhances soil water retention through mechanisms including water absorption and expansion, formation of a binding film, strengthening inter-particle cohesion, and reducing crack width. Among these, decreasing the evaporation surface area of cracks is a key pathway for suppressing the rate of water loss. Therefore, this study uses the variation in desiccation time across four wetting–drying cycles as the basis for evaluating the water-holding capacity of the modified soil.

**Figure 8:**
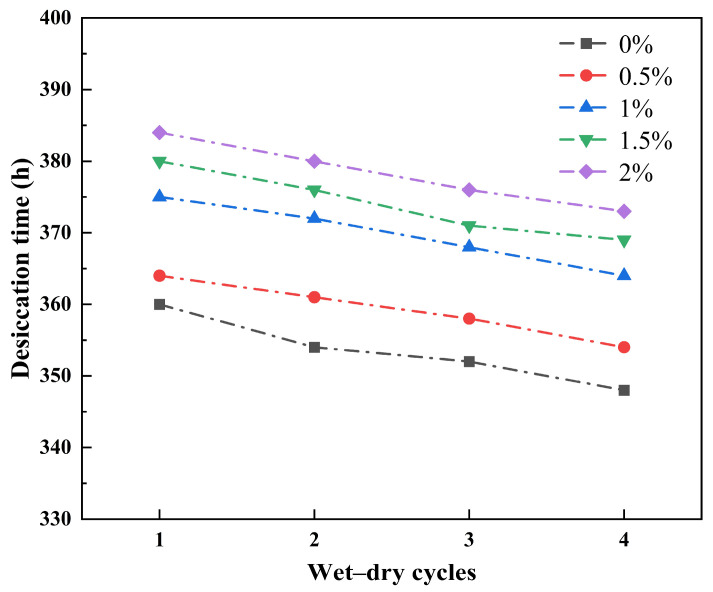
Desiccation time variation with the number of wetting-drying cycles.

As shown in Figure 8, during the first cycle, the dehumidification time increased with the addition of guar gum, reaching 374 h, 379 h, 390 h, 395 h, and 399 h, respectively. This is because guar gum enhances soil water retention and inhibits evaporation rates through mechanisms such as water absorption and expansion, the formation of a binding membrane, and the strengthening of intergranular cohesion. After four freeze–thaw cycles, the desiccation times decreased to 362 h, 368 h, 379 h, 384 h, and 388 h, respectively, indicating an overall reduction in desiccation duration. This indicates that wetting–drying cycles exerted certain effects on soil structure. However, guar gum addition maintained low soil permeability by thickening pore fluids, strengthening intergranular cohesion, and inhibiting the development of drying cracks. Consequently, it delayed the drying process, further enhancing soil water retention and durability. Therefore, the degree of crack development is a key factor affecting the water-holding capacity of the soil, and the crack-resistant effect of guar gum is the core mechanism for delaying water migration.

#### 3.3.2. Effects of Wetting–Drying Cycles on Morphological Parameters

**Figure 9:**
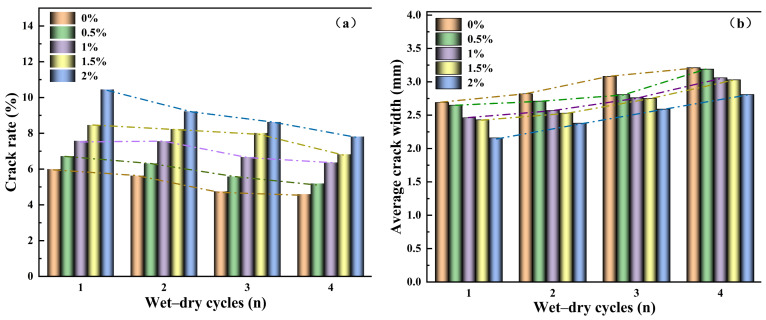
Curves illustrating the relationship between wetting-drying cycle counts and crack parameters. (**a**) Crack rate vs. cycle count, (**b**) Mean crack width vs. cycle count.

To further investigate the evolution patterns of cracks during wetting–drying cycles, four wetting–drying cycle tests were conducted on specimens with different guar gum polymer content levels. Quantitative analysis of the crack images yielded the results shown in Figure 9. The figure indicates that as the number of wetting–drying cycles increases, both the crack density and average crack width of the specimens exhibit an upward trend, ultimately leading to a stable crack network morphology.

Figure 9a reveals the evolution of surface crack rate in silty clay after 1–4 wetting–drying cycles at different guar gum polymer content levels. During the first cycle, the crack rate of all samples with different guar gum polymer contents initially increased rapidly before stabilizing. In subsequent cycles 2–4, the binder film effect delayed crack initiation due to variations in guar gum polymer content. As the number of wetting–drying cycles increased, the crack rates of the native soil during cycles 1–4 were 5.98%, 5.63%, 4.75%, and 4.60%, respectively, representing decreases of 5.85%, 20.57%, and 23.08% compared to the first cycle. Compared to the native soil, using a 1.5% guar gum polymer content as an example, the crack rates during the 1st to 4th cycles were 8.46%, 8.4%, 8.01%, and 6.82%, respectively, representing decreases of 0.71%, 5.32%, and 19.39% compared to the first cycle. The addition of guar gum promotes a more uniform distribution of cracks within the soil matrix. Simultaneously, enhancing soil structure improves the stability and connectivity of the crack network. Overall, specimens with varying guar gum dosages exhibited an initial rapid increase in fissure density followed by gradual stabilization, revealing the cumulative effect of wetting–drying cycles on soil fissure development. Guar gum addition mitigated fissure clustering by improving internal soil structure, thereby promoting a more balanced and stable fissure network.

Figure 9b shows the variation in average crack width between native soil and guar gum polymer-modified soil under different numbers of wetting–drying cycles. After the first wetting–drying cycle, the average crack width of native soil was 2.69 mm. In comparison, the average crack widths of soils modified with different guar gum polymer dosages were relatively minor at 2.65 mm, 2.46 mm, 2.43 mm, and 2.16 mm, respectively. This indicates that guar gum polymer addition suppressed the expansion of crack width in silty clay, with larger guar gum polymer dosages resulting in smaller average crack widths. After the fourth wetting–drying cycle, the average crack width of the untreated soil was 3.21 mm. In comparison, the average crack widths for soils with different guar gum polymer additions were 3.19 mm, 3.06 mm, 3.03 mm, and 2.81 mm, respectively. Compared to the untreated soil, the average crack width decreased by 0.62%, 4.67%, 5.61%, and 12.46% with different guar gum polymer dosages, respectively. This indicates that the average crack width gradually increases with the number of wetting–drying cycles. The incorporation of guar gum polymer effectively mitigated the drying-wetting cycle’s promotion of crack width development in silty clay, maintaining the average crack width within a narrower range. This is primarily attributed to the extension of shorter cracks and their rapid coalescence with other crack networks, ultimately forming wider cracks—a phenomenon consistent with previous studies.

The quantitative analysis results above are consistent with the experimental observations described earlier. It can be concluded that the gelatinous film formed by guar gum polymer during the wetting stage promotes the formation of ‘healing ridges. As structural weak points, these ridges induce earlier crack initiation while simultaneously encouraging the development of multiple cracks.

#### 3.3.3. Effect of Wetting–Drying Cycles on the Connectivity Coefficient of Crack Networks

The connectivity coefficient (K) of a crack network is defined by the number of intersections (I) and endpoints (E). It serves as a crucial indicator reflecting the structural stability and water transport capacity of the crack network, characterizing the reversible connectivity changes of cracks during wetting–drying cycles. This coefficient reveals the dynamic evolutionary characteristics of the crack network, from expansion to closure and subsequent regeneration. The calculation formula is as follows:

(1) $$K=\frac{I}{E+I}$$

In the formula, I denotes the number of intersection points; E denotes the number of endpoints; K is a constant with a range of 0 ≤ K ≤ 1. When K equals 0, all cracks are independent and do not form a network. When K approaches 1, most cracks are interconnected to form a complex network. A larger K value indicates better connectivity and water conductivity within the crack network.

**Figure 10:**
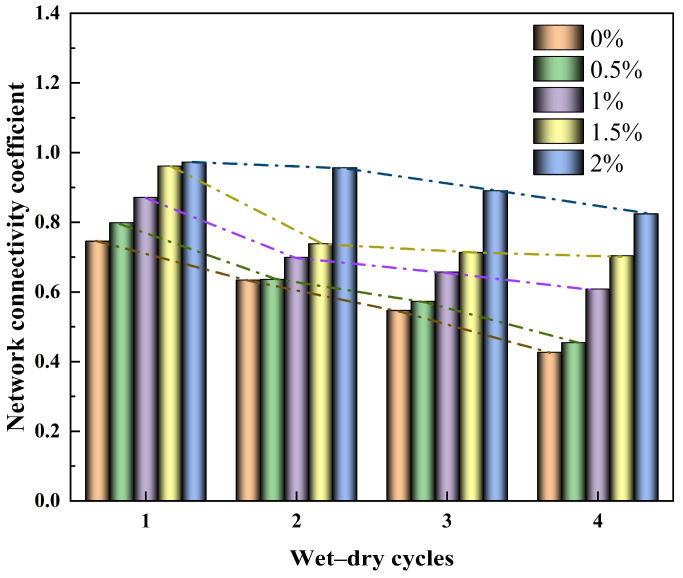
Variation curve of crack network connectivity coefficient in modified soil under wetting–drying cycles with different guar gum polymer dosages.

Figure 10 illustrates the evolution of the connectivity coefficient K of the crack network with the number of wetting–drying cycles. Overall, the connectivity coefficient of the crack network gradually decreases with increasing cycle number for both native soil and soils modified with different guar gum polymer contents. This indicates that repeated wetting–drying cycles cause the crack network to undergo a dynamic developmental process of expansion, closure, and regeneration. During the first two cycles, crack expansion and overlap enhanced network connectivity. However, in later cycles, partial closure or healing of fine cracks led to simultaneous reductions in both junction and endpoint counts, with a more pronounced decrease in junction counts, resulting in an overall decrease in connectivity coefficient. This demonstrates that the cycling process not only alters crack quantity and geometry but also reshapes the spatial structure of the crack system. Under identical cyclic conditions, the crack connectivity coefficient exhibited an upward trend with increasing guar gum polymer content, indicating that guar gum polymer addition enhances the structural stability and reversible connectivity of the crack network. This is primarily attributed to the flexible film layer formed by guar gum polymer on crack wall surfaces, which preserves potential connection pathways even after crack closure, facilitating easier reopening during subsequent cycles. Therefore, the connectivity coefficient K reflects the evolution of the crack network and water transport capacity in modified silty clay during wetting–drying cycles, serving as a crucial indicator for evaluating the complexity of the crack structure and its moisture response behaviour.

#### 3.3.4. Effect of Wetting–Drying Cycles on the Crack Shape Factor

Shape factor and fractal dimension jointly characterize the geometric complexity and morphological evolution of crack networks. The former describes the regularity and roughness of crack-segmented blocks, while the latter reflects the heterogeneity of crack branching structures. Their combined variations reveal the structural evolution of crack networks from complexity to simplicity and from instability to stability under repeated wetting–drying cycles.

(1) Shape factor

The shape factor, f_f_, and average shape factor, f_fa_, can be used to describe the roughness of crack block geometry, with values calculated from the block’s area and perimeter. The shape factor characterizes the roundness of block boundaries, ranging from 0 to 1. For a perfect circle, the shape factor is 1; for a perfect square, it is 0.785.

(2) $$f_{f}=\frac{4\pi S}{C^{2}}$$

(3) $$f_{\mathrm{fa}}=\frac{\sum_{i=1}^{N}\frac{4\pi S_{i}}{C_{i}^{2}}}{N}$$

In Equations (2) and (3): S—area; C—perimeter (mm); N—total number of blocks.

**Figure 11:**
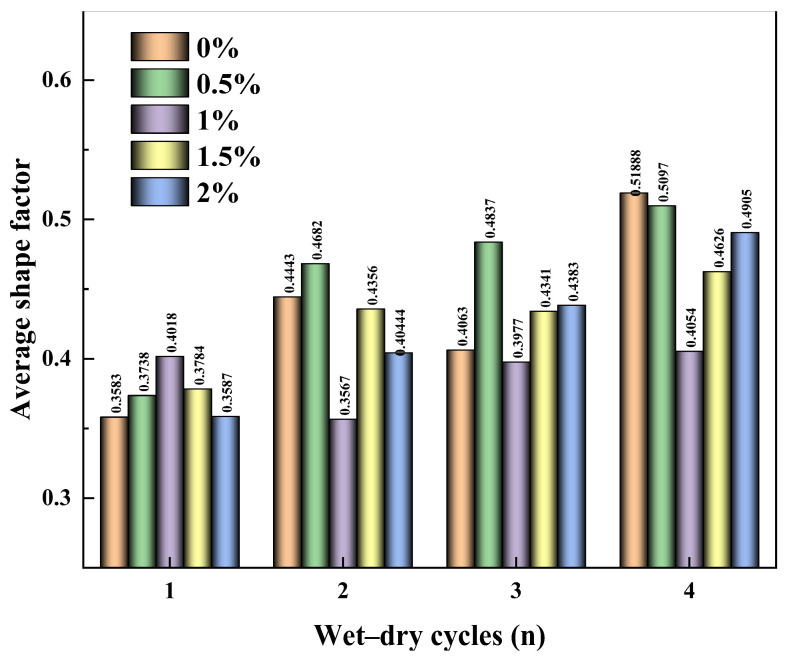
Relationship between wetting–drying cycles and mean crack shape factor.

As shown in Figure 11, with increasing wetting–drying cycles, the mean shape factor of soil blocks in both plain soil and guar gum polymer-modified soils fluctuated within a narrow range. Since the wetting–drying effect had little influence on the mean shape factor of the blocks for both plain and guar gum polymer-modified soils, the mean values were directly adopted for analysis of each group of specimens. As illustrated in Figure 8, the shape factors of blocks in both plain soil and guar gum polymer-modified soils showed little difference, ranging from 0.31 to 0.57. This indicates that the soil blocks of both plain soil and guar gum polymer-modified soils were predominantly irregular polygons.

(2) Fractal dimension

The fractal dimension of cracks is a key parameter characterizing the complexity of crack networks [30]. A higher value indicates more developed cracks and a more intricate morphology. To reveal the relationship between fractal dimension and the number of wetting–drying cycles at different guar gum polymer content levels, nonlinear fitting was performed on the area fractal dimension and the length fractal dimension:

(4) $$D_{f,area}=A_{1}\exp(-\frac{n}{B_{1}})+C_{1}$$

(5) $$D_{f,length}=A_{2}\exp(-\frac{n}{B_{2}})+C_{2}$$

In the formula, *D_f_*, area is the area fractal dimension, *D_f_*, length is the length fractal dimension, and n is the number of wetting–drying cycles. The values of the relevant parameters A1, B1, C1, A2, B2, and C2 are shown in Table 3.

**Figure 12:**
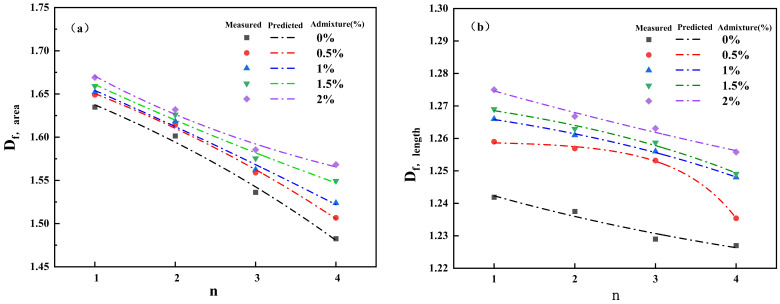
Response of fractal dimensions to cyclic wetting-drying. (**a**) Evolution of crack area dimension, (**b**) Evolution of crack length dimension.

As shown in Figure 12, both the fractal dimension of crack area and the fractal dimension of crack length exhibit a decreasing trend with increasing wetting–drying cycles, with R^2^ > 0.94, indicating excellent model fitting performance. During the fitting process, the A and C values of specimens with different guar gum polymer content showed minimal variation. In contrast, the B value exhibited significant fluctuations across varying guar gum polymer levels. This indicates that guar gum polymer inhibits the rate of crack development, suggesting a gradual reduction in the complexity of the crack network toward regularization.

Changes in shape coefficient and fractal dimension reveal that the crack network evolves from complexity toward simplified stabilization under repeated wetting–drying cycles. As cycle frequency increases, crack blocks exhibit increasingly regular shapes, fractal dimension decreases, and network complexity diminishes. These changes correlate with the decline in connectivity coefficient, indicating that guar gum polymer-modified soil undergoes a dynamic process of expansion, healing, regeneration, and stabilization under cyclic loading, ultimately achieving crack structural stability.

#### 3.3.5. Effect of Wetting–Drying Cycles on Crack Depth Rate

The crack depth ratio is a quantitative measure of crack longitudinal expansion and is a critical parameter for soil crack resistance, stability, and structural integrity. It reflects the inhibitory effect of the guar gum polymer on the longitudinal development and depth accumulation of cracks, thereby revealing the modified soil’s longitudinal crack-resistance performance under wetting–drying cycles. After the fourth desiccation, the maximum crack depth of the soil was measured using a high-precision digital depth gauge with a fine rod (resolution: 0.001 mm). The calculation formula for the crack depth ratio is as follows:

(6) $$R_{d}=\frac{d_{\max}}{h}\times 100\%$$

In Equation (6): hmax, maximum crack depth of the specimen (mm); h is the crack height of the specimen after the fourth wetting–drying cycles (mm).

**Figure 13:**
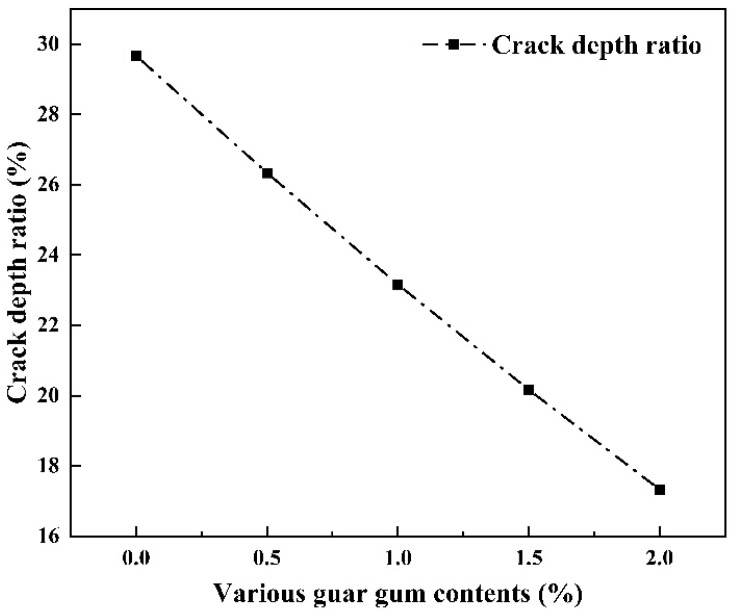
Relationship between crack depth ratio and wetting–drying cycles.

As shown in Figure 13, the graph depicts the relationship between the crack depth ratio and guar gum content for the specimens. As the guar gum content increased, the crack depth ratio of the specimens decreased gradually. The ratios for the plain soil and specimens with different guar gum contents were 29.66%, 26.33%, 23.16%, 20.16%, and 17.33%, respectively, showing a significant overall reduction. These results indicate that guar gum effectively inhibits longitudinal crack propagation through two primary mechanisms: upon swelling, guar gum forms a continuous adhesive film on particle surfaces, filling pores and enhancing interfacial cohesion between particles, thereby improving overall structural integrity; guar gum’s water absorption and expansion mitigate internal soil stress differences, reducing stress concentration at crack tips. Consequently, the reduction in crack depth ratio reflects that guar gum-modified silt loam exhibits superior crack resistance and shape retention under wet-dry cycling. This demonstrates that the formation of the guar gum film significantly enhances the material’s durability.

## 4. Analysis and Discussion

### 4.1. Microstructural Characteristics and Improvement Mechanisms

Existing research indicates [34] that, with the progression of wetting–drying cycles, the original aggregates in the soil gradually break down, forming a disordered, homogeneous, and loose structure, which leads to a continuous increase in total porosity. Therefore, this section focuses on SEM measurements of undisturbed soil and 1% guar gum polymer-modified soil that have not undergone wetting–drying cycles.

**Figure 14:**
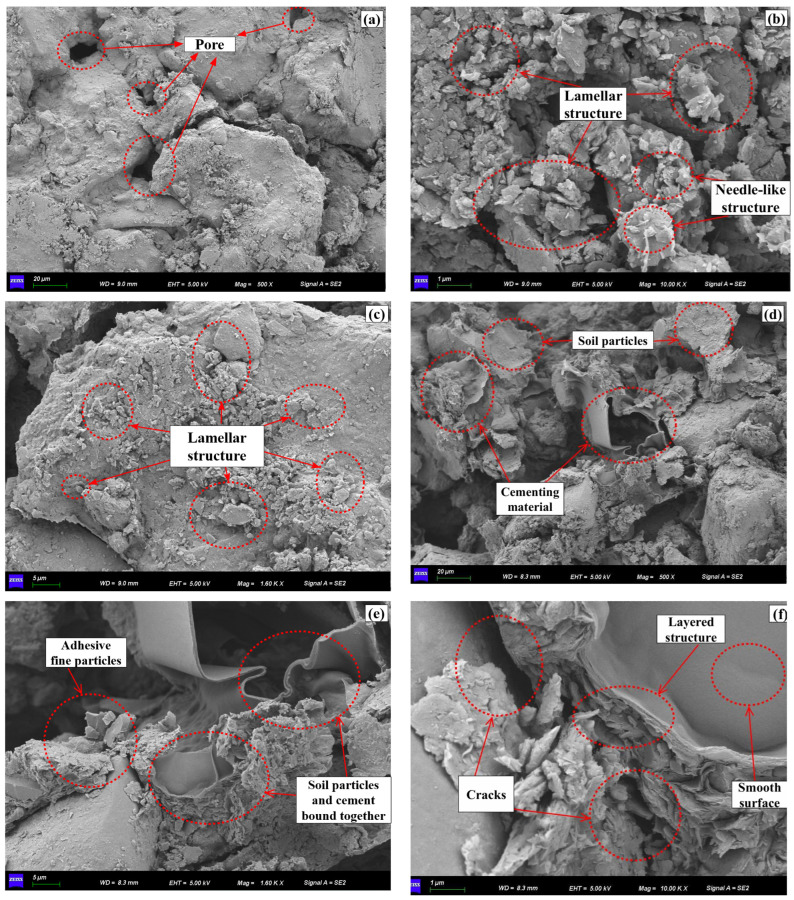
Microstructures of guar gum polymer-modified silty clay. (**a**) Plain soil at 500× magnification, (**b**) Plain soil at 2000× magnification, (**c**) Plain soil at 10,000× magnification, (**d**) 1% guar gum polymer-modified soil at 500× magnification, (**e**) 1% guar gum polymer-modified soil at 2000× magnification, (**f**) 1% guar gum polymer-modified soil at 10,000× magnification.

The microstructure of soil is a key factor influencing crack formation. To investigate the effect of guar gum on soil microstructure, this study employed scanning electron microscopy (SEM) to analyze the microstructure of both unmodified soil and soil modified with 1% guar gum, with results shown in Figure 14. Analysis indicates that the particle morphology of the unmodified soil samples, as depicted in Figure 14a–c, primarily exhibits needle-like and dispersed flake structures. Such structures are inherently unstable, containing numerous voids that readily form cracks during drying. Compared with the native soil, Figure 14d–f reveal that the surface of the guar gum-modified soil is relatively smooth, with no obvious voids. soil particles are enveloped and interconnected by unstable lamellar structures, forming more stable aggregates. A uniform cementation layer covers the particle surfaces, tightly bonding the soil particles into a cohesive whole. This effectively agglomerates fine particles, reducing the number of inter-particle voids and the connectivity of larger pores, thereby forming a dense ‘bonded interlocking’ structure (indicated by the arrow in Figure 14d).

There exists a clear relationship between the aforementioned microstructural characteristics and macro-fracture behaviour. The continuous cementation membrane formed by guar gum (Figure 14d) effectively enhances inter-particle cohesion, directly explaining the lower average fissure width in modified soil compared to undisturbed soil (Figure 9b). Concurrently, the ‘bonded bridging’ structure (Figure 14e) inhibits longitudinal fissure expansion by constraining particle displacement, consistent with results under fissure depth rate shown in Figure 13. The encapsulation of the cementation film observed under high-power microscopy (Figure 14f) reveals, at the microscopic level, the mechanism behind the stabilization of the fracture network connectivity coefficient (Figure 10). The cementation film maintains structural continuity during the wetting phase, enabling fractures to regenerate along the original ‘healing ridges’.

In summary, the granular bridging structures observed in SEM images of guar gum quantitatively explain, at the microscopic level, the macroscopic phenomena of reduced crack width, suppressed crack depth, and stabilized network. By mitigating the concentration of drying shrinkage stresses, this mechanism effectively inhibits crack propagation, resulting in finer and denser cracks. Consequently, the soil’s crack-resistance properties are further enhanced.

Furthermore, as a natural biopolymer, guar gum can enhance the crack resistance and structural stability of soil. However, its biodegradability, typically driven by microbial activity, may lead to gradual alterations in its physicochemical properties during prolonged use. This could consequently affect its stability within the soil matrix and its influence on soil structural stability, long-term performance, and crack resistance. The experimental period of this study was relatively brief, primarily focusing on the initial ameliorative effects of guar gum during the wet-dry cycling phase. It did not quantitatively analyze its long-term biodegradation process or changes in durability.

**Figure 15:**
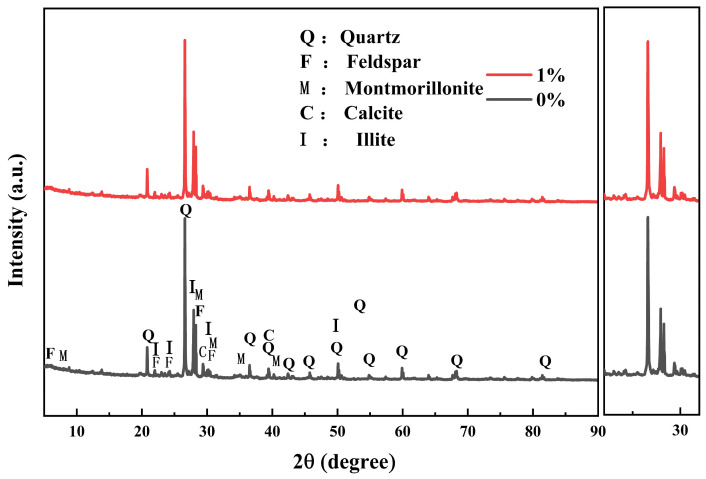
X-ray Diffraction Patterns of Silty Clay with Varying Guar Gum Polymer Content.

X-ray diffraction (XRD) analysis of mineral changes before and after guar gum incorporation, as shown in Figure 15, reveals that the primary mineral constituents of the siliceous clay comprise quartz, feldspar, montmorillonite, calcite, and illite. Comparison of XRD patterns for siliceous clay with varying guar gum content indicates that the incorporation of guar gum did not generate new diffraction peaks, nor did the peak intensities exhibit significant alterations. This indicates that no obvious chemical reactions involving mineral synthesis or decomposition were observed between guar gum and the silty clay. Calculations based on Bragg’s law determined the interlayer spacing of montmorillonite crystals in the silty clay before and after guar gum incorporation. The interlayer spacing of montmorillonite crystals remained unchanged, indicating no ion exchange occurred between guar gum and the siliceous clay. It should be noted that XRD is primarily used to identify crystalline mineral phases; it is challenging to thoroughly investigate the amorphous guar gum polymer, its adsorption state on soil particle surfaces, or chemical interactions (such as hydrogen-bond formation). Consequently, XRD results cannot directly confirm the chemical adsorption of guar gum, representing a methodological limitation of this study. Future research may employ surface analysis techniques such as Fourier Transform Infrared Spectroscopy (FTIR) or X-ray Photoelectron Spectroscopy (XPS) to investigate further potential intermolecular interactions at the guar gum-soil particle interface.

(7) nλ=2dsinθ

In Equation (7): n: Diffraction order, typically an integer value, usually set to 1. λ: X-ray wavelength (unit: nm), commonly Cu-Kα with a wavelength of 0.154060 nm. d: Interplanar spacing (unit: nm), representing the distance between adjacent crystal planes. θ: Diffraction angle (unit: degrees, °), the angle between the incident X-ray and the normal to the crystal plane.

### 4.2. Crack Development Analysis

**Figure 16:**
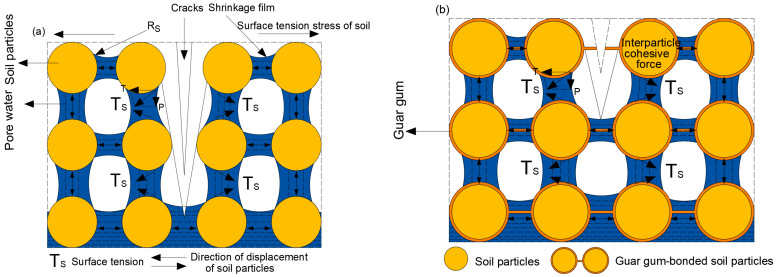
Mechanism of Guar Gum Polymer Inhibiting Crack Development in Silty Clay. (**a**) Silty clay, (**b**) Guar gum polymer-modified silty clay.

Figure 16 illustrates the mechanism of fissure formation. In a naturally saturated state, soil pores are filled with water, forming a hydration film on particle surfaces that creates a particular spacing between particles. During drying, capillary water action forms a shrinkage film. Its surface tension (TS) decomposes into lateral tensile stress T and vertical compressive stress P acting upon soil particles. This forces particles to converge laterally and consolidate vertically, reducing pore volume and causing soil shrinkage. Matrix suction [35] is generally defined as the difference between the air pressure US and the capillary water pressure UW. These pressures relate to the surface tension TS of the contraction film as follows:

(8) $$\psi =U_{S}-U_{w}=\frac{2T_{S}\cos\theta }{r}=\frac{2T_{S}}{R_{S}}$$

In Equation (8): θ is the angle of capillary water, r is the radius of the capillary pore, R_S_ is the radius of curvature of the shrinkage film, and T_S_ is the surface tension of the shrinkage film.

During soil shrinkage, soil particles simultaneously experience the effects of matrix cohesion and surface tension, which limit contraction and consequently generate tensile stresses at the soil surface. As moisture evaporates during drying, the capillary curvature radius in the ‘healing ridge’ region progressively diminishes. This reduces the spacing between soil particles, causing them to converge and thereby increasing inter-particle forces. Due to variations in inter-particle cohesive strength and uneven water evaporation rates, tensile stresses within the soil are distributed non-uniformly, resulting in stress concentration. When the tensile stress at a particular point exceeds the cohesive strength between soil particles, cracks will form.

Guar gum molecules can form hydrogen bonds with water molecules, leading to hydration swelling. The molecular chains unwind and disperse uniformly, forming a viscous colloidal solution upon dissolution in water. On the one hand, this solution fills the interstitial spaces between soil particles [36], altering the soil’s internal pore structure and slowing water migration, thereby reducing tensile stresses between soil particles. Concurrently, during this filling process, colloidal particles form a layered structure on the surfaces of soil particles. This enveloping action prevents direct contact between water and clay minerals (such as montmorillonite), thereby reducing the thickness of the hydration film on the mineral surfaces. As the guar gum-modified soil gradually dries and loses water through evaporation, the interfacial layer on the soil particle surfaces becomes progressively concentrated. This ultimately forms a cementing layer that enhances soil stability (as shown in Figure 14d). This layer effectively prevents direct contact between soil particles and moisture during subsequent rewetting, particularly inhibiting the hydration expansion of clay minerals. Consequently, it reduces the degree of swelling and shrinkage in silty clays [37] and suppresses crack propagation during the fourth cycle.

In summary, guar gum effectively stabilizes the fissure network structure through multiple wet-dry cycles by absorbing water and swelling, forming a cohesive film, and filling pores. This mechanism provides a theoretical basis for subsequent quantitative analysis of crack resistance and healing performance (as illustrated in Figure 16b).

## 5. Conclusions

This study investigated the development, healing, and morphological stability patterns of fissure networks in guar gum-modified soils over four natural wet-dry cycles, using digital image processing and microstructural analysis. Results indicate that during the first wet-dry cycle, cracks in the unmodified soil evolved from “fine cracks” to “wide primary cracks,” while cracks in the guar gum-modified soil gradually transitioned from “T-shaped” to “Y-shaped” and progressively healed during the wetting process, forming “healing ridges.” During subsequent cycles 2–4, cracks largely regenerated at original locations, though their development directions varied during each drying phase.

As the number of wet-dry cycles increased, the complexity of the fracture network decreased, with reduced fracture density and fractal dimension, while the average fracture width increased. The addition of guar gum effectively reduced the fracture depth ratio, and both the connectivity coefficient and shape coefficient tended toward stability. This indicates that guar gum effectively enhances the connectivity and morphological stability of the fracture network, thereby improving the durability of the modified soil. Microscopic findings using scanning electron microscopy (SEM) confirmed that guar gum promotes the formation of “bonded bridging” structures between soil particles. This mechanism fills pores and enhances interparticle cohesion, effectively inhibiting crack width expansion. Furthermore, X-ray diffraction (XRD) analysis indicated that the addition of guar gum did not induce chemical changes in the soil’s mineral composition.

In summary, guar gum enhances soil stability through dual mechanisms of physical filling and film-forming crack inhibition, reduces crack width, and guides orderly crack regeneration along “healing ridges.” This improves the long-term stability of modified soil under freeze–thaw cycles. This study provides crucial material parameters and theoretical support for ecological protection of silty clay slopes, demonstrating the potential for large-scale application of guar gum-modified soil in slope engineering.

## Figures and Tables

**Table 1:** Basic physical parameters and particle size distribution data of soil.

Liquid Limit/%	Plastic Limit/%	Plasticity Index	Optimal Moisture Content/%	Maximum Dry Density/g·cm^3^	Particle Size Analysis/%(mm)				
27	16.9	10.1	18.1	1.85	5~1	1~0.5	0.5~0.25	0.25~0.075	<0.075
					5.16	14.31	6.13	55.82	18.58

**Table 2:** Meteorological characteristics of four wetting-drying cycles.

W–D Cycle	Period	Season	Meteorological Summary
1	17 July 2024–2 August 2024	Midsummer	High temperatures and low humidity
2	28 August 2024–13 September 2024	Late summer	Marked fluctuations in temperature and humidity
3	22 September 2024–8 October 2024	Autumn	Cool and windy
4	17 October 2024–2 November 2024	Early winter	Low air temperature and strong winds

**Table 3:** Fitting parameters for fractal dimension models.

Guar Gum Polymer/%	A1	A2	B1	B2	C1	C2	Rarea2	Rlength2
0	−0.45205	0.01746	5.59745	−5.31364	1.85871	1.20528	0.977	0.960
0.5	−0.42484	−0.09223	5.76876	0.73348	1.86281	1.25902	0.992	0.995
1	−0.97958	−0.04194	19.59073	3.72309	2.45276	1.28016	0.975	0.998
1.5	0.36462	−0.04324	−11.87996	3.2202	1.15046	1.28104	0.963	0.967
2	0.6936	0.05848	−8.7204	−11.81797	1.47589	1.19259	0.959	0.949

Note: A1, B1, C1: area fractal dimension fitting parameters; A2, B2, C2: length fractal dimension fitting parameters; Rarea2, Rlength2: goodness of fit.

## Data Availability

The original contributions presented in this study are included in the article. Further inquiries can be directed to the corresponding authors.
